# A new small-angle X-ray scattering model for polymer spherulites with a limited lateral size of the lamellar crystals

**DOI:** 10.1107/S2052252519011035

**Published:** 2019-08-31

**Authors:** Xiang-Yang Li, Jian-Jun Ding, Yan-Ping Liu, Xing-You Tian

**Affiliations:** a Institute of Applied Technology, CAS Key Laboratory of Photovoltaic and Energy Conservation Materials, Hefei Institutes of Physical Science, Chinese Academy of Sciences, Hefei, Anhui 230088, People’s Republic of China; b National Center for International Research of Micro–Nano Molding Technology and Key Laboratory for Micro Molding Technology of Henan Province, Zhengzhou University, Zhengzhou 450002, People’s Republic of China

**Keywords:** small-angle X-ray scattering, evanescent waves, lamellar thickness, spherulite

## Abstract

As is well known, polymers commonly form lamellar crystals, and these assemble further into lamellar stacks and spherulites during quiescent crystallization. Fifty years ago, Vonk and Kortleve constructed the classical small-angle X-ray scattering theory (SAXS) for a lamellar system, in which it was assumed that the lamellar stack had an infinite lateral size [Vonk & Kortleve (1967 ▸), *Kolloid Z. Z. Polym.* **220**, 19–24]. Under this assumption, only crystal planes satisfying the Bragg condition can form strong scattering, and the scattering from the lamellar stack arises from the difference between the scattering intensities in the amorphous and crystalline layers, induced by the incident X-ray beam. This assumption is now deemed unreasonable. In a real polymer spherulite, the lamellar crystal commonly has dimensions of only a few hundred nanometres. At such a limited lateral size, lamellar stacks in a broad orientation have similar scattering, so interference between these lamellar stacks must be considered. Scattering from lamellar stacks parallel to the incident X-ray beam also needs to be considered when total reflection occurs. In this study, various scattering contributions from lamellar stacks in a spherulite are determined. It is found that, for a limited lateral size, the scattering induced by the incident X-ray beam is not the main origin of SAXS. It forms double peaks, which are not observed in real scattering because of destructive interference between the lamellar stacks. The scattering induced by the evanescent wave is the main origin. It can form a similar interference pattern to that observed in a real SAXS measurement: a Guinier region in the small-*q* range, a signal region in the intermediate-*q* range and a Porod region in the high-*q* range. It is estimated that, to avoid destructive interference, the lateral size needs to be greater than 11 µm, which cannot be satisfied in a real lamellar system. Therefore, SAXS in a real polymer system arises largely from the scattering induced by the evanescent wave. Evidence for the existence of the evanescent wave was identified in the scattering of isotactic polypropyl­ene. This study corrects a long-term misunderstanding of SAXS in a polymer lamellar system.

## Abstract 2

Small-angle X-ray scattering in real polymer systems arises largely from the scattering induced by the evanescent wave. Evidence for the existence of the evanescent wave has been identified in the scattering from isotactic polypropylene.

## 1. Introduction   

In recent decades polymers have been extensively employed in numerous fields, for example mobile phones, computers, cars, aircraft and so on. According to statistics, global plastic production amounted to 311 million tonnes in 2014, two thirds of which were semi-crystalline polymers (Zalasiewicz *et al.*, 2016 ▸). As is well established, polymers form folded-chain lamellar crystals and these assemble further to form lamellar stacks and spherulites during quiescent crystallization (Li *et al.*, 2001 ▸). The lamellar thickness and the long period influence strongly the mechanical, thermodynamic and other properties of the bulk materials (Strobl, 2000 ▸; Ma *et al.*, 2009 ▸). Thus, their accurate determinations have been key to revealing the relationship between structure and properties. Generally, they can be determined by atomic force microscopy (AFM) (Mullin & Hobbs, 2011 ▸; Savage *et al.*, 2015 ▸; Wang *et al.*, 2018 ▸) and transmission electron microscopy (TEM) (Rastogi *et al.*, 1997 ▸; Maiti *et al.*, 2000 ▸; Yamada *et al.*, 2003 ▸) in real space, or small-angle X-ray scattering (SAXS) in reciprocal space (Strobl & Schneider, 1980 ▸; Hashida *et al.*, 2010 ▸; Wang *et al.*, 2014 ▸). Compared with AFM and TEM, SAXS has been the most powerful tool to observe structural evolution on the nano­scale, due to its higher time-resolving capability and lower requirements for sample preparation.

To obtain structural information from SAXS, it is first necessary to know where the scattering is from and then an effective method can be identified. According to general SAXS theory, to determine the scattering from an object at a wavevector *A_q_*, we need the scattering amplitude *A_i_* and electron density η_*i*_ at every point in this object (Guinier & Fournet, 1955 ▸),
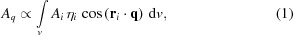
where the dot product of the position vector **r**_*i*_ and the wavevector **q** is the phase of the scattered X-rays (Jeu, 2016 ▸). Determining the scattering from a crystallized polymer seems to be a huge task, since it is necessary to know all the information on the lamellar stacks within the system, *e.g.* orientation, number, lateral size, thickness and so on.

**Figure 1:**
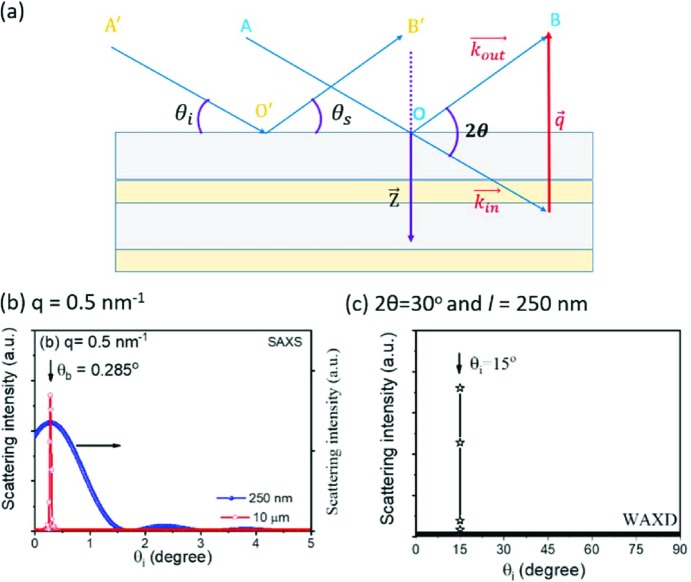
(*a*) The scattering from a lamellar stack. (*b*) and (*c*) The single-crystal plane scattering intensities at different incident angles under fixed *q* or 2θ. In (*b*), *q* is fixed at 0.5 nm^−1^, while 2θ is fixed at 30° in (*c*). The lateral size in (*b*) is assumed to be 250 nm or 10 µm, while it is assumed to be 250 nm in (*c*). The average distance between adjacent electrons and the wavelength of the X-rays are assumed to be 0.17 nm and 0.124 nm, respectively.

Fifty years ago, Vonk and Kortleve applied SAXS theory to a polymer lamellar system (Vonk & Kortleve, 1967 ▸). To simplify the computation, they assumed that the lamellar stack had an infinite lateral size and numerous lamellae, such that the lamellar stack can be reduced to a one-dimensional two-phase structure. Further, they assumed that the scattering was mainly from the lamellar stack satisfying the Bragg condition, which can be seen from their expression for scattering *i*(*q*),
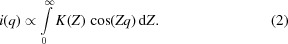
In this expression, the phase difference is written as *Zq*, where the vectors **Z** [the distance between parallel (00*l*) planes in the lamellar stack] and **q** have been changed to scalars. It can be written in such a form only when the position vector **Z** is parallel to the wavevector **q**, otherwise it should be written as *qZ*cosα, where α is their intersection angle. When **Z** is parallel to **q**, the crystal plane inevitably has the same intersection angles with the incident and scattered X-rays (θ_i_ = θ_s_), as seen in Fig. 1 ▸(*a*). This is actually the Bragg condition. Under this condition, scattered X-rays from the crystal plane have the same phase, interfering constructively to give strong scattering. Therefore, *i*(*q*) should be the scattering from the lamellar stack satisfying the Bragg condition. Under this assumption, the scattering is only determined as a correlation function *K*(*Z*), which is defined as follows (Vonk & Kortleve, 1967 ▸): 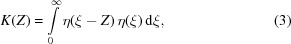
where η(ξ) and η(ξ − *Z*) are the electron densities at positions ξ and ξ − *Z*, respectively. This equation describes the statistical correlation between the electron densities at two arbitrary points separated by a fixed distance, carrying structural information on the lamellar stack.

The latter assumption is reasonable for an infinite lateral size, which can be seen from the contributions of crystal planes with different intersection angles to the scattering at a given wavevector. Assuming that the lateral size of the lamellar crystal is 10 µm, the distance between adjacent electrons is 0.17 nm and the wavelength of the X-rays is 0.124 nm, it will be found that only the crystal plane with θ_i_ = 0.28° makes a significant contribution to the scattering at *q* = 0.5 nm^−1^ [see Fig. 1 ▸(*b*)], which corresponds to a scattering angle of exactly 0.28°. A detailed determination is given in the supporting information.

Based on the above assumption, Vonk & Kortleve (1967 ▸) further assumed that the overall scattering intensity from a lamellar system *I*(*q*) can be determined as follows: 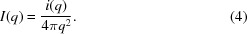
Their argument had two points: (i) the scattering intensity is concentrated in a narrow range satisfying the Bragg condition, and (ii) in a lamellar system, there are differently orientated lamellar stacks, for which the intersection angles with **q** are distributed evenly between 0° and 360°. One can imagine that the wavevector **q** forms a sphere in reciprocal space. The probability of forming Bragg scattering is only 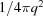. Therefore, the average intensity or said real scattering intensity is only 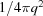 of *i*(*q*). Conversely, if the real intensity is known, one can obtain the scattering of the lamellar stacks satisfying the Bragg condition,
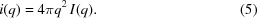
This is the so-called Lorentz correction (Vonk & Kortleve, 1967 ▸).

However, in a real lamellar system, the lateral size is not so long, but rather is commonly only a few hundred nanometres (Basire & Ivanov, 2000 ▸; Li *et al.*, 2001 ▸; Hobbs *et al.*, 2001 ▸; Lei *et al.*, 2002 ▸, 2003 ▸; Mullin & Hobbs, 2011 ▸; Liu *et al.*, 2013 ▸; Ono & Kumaki, 2018 ▸). At such a limited lateral size, is the Bragg condition still the necessary condition for strong scattering? Let us look again at the scattering from the crystal planes with different incident angles at 0.5 nm^−1^, but with the lateral size changed to 250 nm. Unlike crystal planes with a larger lateral size, crystal planes in a broad intersection angle range (0 ≤ θ_i_ < 1°) make significant contributions to the scattering [see equation (1)]. This means that numerous lamellar stacks in a spherulite make contributions to the scattering at a given wavevector. Equation (4) is thus no longer valid. The interference of the scattering contributions from these lamellar stacks must be considered.

In addition, at θ_i_ = 0 the scattering intensity is not equal to 0 [see Fig. 1 ▸(*b*)]. At this point, will scattering arise from a lamellar stack parallel to the incident X-ray beam? As is known, when θ_i_ = 0 the incident X-rays will not enter the lamellar stack but will be totally reflected. Half a century ago, one could assume confidently that if total reflection occurred, no SAXS would be observed. The reason was that when total reflection occurred, no X-rays entered the lamellar stack and thus no scattering could be induced from electrons below the first interface. In 1971, Schultz performed an interesting calculation (Schultz, 1971 ▸). It was found that, when the polymer lamellar spacing was larger than 90–100 nm, the Bragg angle θ_b_ (2*Z*sinθ_b_ = λ) could be smaller than the critical total reflection angle. Combining the above assumptions, it was inferred that, at such a high lamellar spacing, SAXS would be replaced by total reflection.

This assumption is in fact wrong since it overlooks the existence of the evanescent wave. Though the incident X-rays cannot enter the lamellar stack, the evanescent wave formed at the first interface can enter and induce scattering from the electrons within the lamellar stack. Furthermore, if these scattered X-rays can interfere constructively, they can also form a strong SAXS signal. Grazing-incidence small-angle X-ray scattering (GISAXS) is formed under exactly such a mechanism (Jeu, 2016 ▸). In the construction of SAXS theory, no one realized the role of the evanescent wave. The first GISAXS result was reported 22 years after the construction of the classical SAXS theory (Levine *et al.*, 1989 ▸; Vonk & Kortleve, 1967 ▸).

Nevertheless, even after the discovery of GISAXS, the scattering induced by the evanescent wave did not receive attention in the scattering from a bulk polymer. One assumed that, even if total reflection occurred, the lamellar stacks involved in total reflection were extremely small because of the smaller density contrast between the amorphous and crystalline layers. Such small lamellar stacks would not change the scattering significantly. Indeed, the lamellar stacks at total reflection are small. According to our estimation, the critical total reflection angle 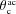 for the amorphous/crystalline interface of isotactic polypropyl­ene (iPP) is around 0.034° when λ = 0.124 nm (see supporting information), much smaller than the value at the polymer/vacuum interface (0.12°) (Sakai *et al.*, 2005 ▸). This means that, if one lamellar stack is involved in total reflection, there will be thousands of lamellar stacks that will be passed through by the incident X-rays directly. Nevertheless, it should not be omitted lightly without careful consideration.

The above features do not exist in wide-angle X-ray diffraction (WAXD). For the same crystal plane with a lateral size of 250 nm, only the crystal plane with θ_i_ = 15° makes a significant contribution to the scattering at 2θ = 30° [see Fig. 1 ▸(*c*)]. This means that it is not necessary to consider the interference between lamellar stacks and the scattering induced by the evanescent wave. In the construction of the classical SAXS theory for lamellar systems, no attention was paid to the difference between WAXD and SAXS and it was still assumed that the scattering arose mainly from the lamellar stacks satisfying the Bragg condition. The description of the classical theory for SAXS of a lamellar system is not complete, lacking the features of SAXS in such a system.

In this study, we will describe scattering in the small-*q* range again, with iPP as an example. The scattering induced by the evanescent wave and that induced directly by the incident X-rays will be described separately. In the description of the scattering induced by the evanescent wave, it will be further divided into two parts, the scattering from interfacial electrons and the scattering from bulk electrons. Their main difference is that a half-wave loss exists in the scattering of interfacial electrons but not in the scattering of bulk electrons. As is well known, reflected light has a half-wave loss when light within an optically less dense medium reflects at an interface in grazing incidence or in normal incidence (Born & Wolf, 1999 ▸), and this will also occur for X-rays. Nevertheless, for X-rays the amorphous phase will be the optically dense medium compared with the crystalline phase, which is the opposite to what happens with light. The X-rays scattered by interfacial electrons have also a half-wave loss, since the reflected X-ray beam is the summation of scattered X-rays. For the scattering from bulk electrons, the half-wave loss does not exist, because the scattering from bulk electrons is related to the refracted X-rays and the refracted X-ray beam always has the same phase as the incident X-rays at the interface (Born & Wolf, 1999 ▸).

To avoid omitting any significant scattering, scattering intensities from all orientated lamellar stacks will be determined first. Then all the scattering induced by incident X-rays will be summed in a spherulite and compared with the scattering induced by the evanescent wave, such that the real origin of the SAXS signal can be identified. For an arbitrarily orientated lamellar stack, we will first determine the scattering of the (00*l*) crystal plane 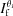 and the interference intensity of parallel crystal planes 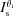. Next, the scattering intensity from the lamellar stack can be determined with their product: 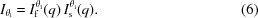
Finally, the real scattering from iPP obtained during isothermal crystallization at a lower temperature will be employed to test the new theory. It has been reported that, at a lower temperature, iPP can form a lamellar two-phase structure (Zhu *et al.*, 2001 ▸; Crist & Schultz, 2016 ▸).

## 2. Materials and method   

The iPP sample employed in this study has a weight-average molecular weight of 720 kg mol^−1^ and a polydispersity of 4.8; it was kindly supplied by SABIC-Europe. The sample as supplied consisted of an iPP film with a thickness of 100 µm. To test our new scattering model, two dimensional (2D) SAXS patterns of the iPP film were obtained during isothermal crystallization at 130°C, using the synchrotron SAXS station equipped with a Mar165 CCD detector on beamline BL16B of the Shanghai Synchrotron Radiation Facility. Thermal history was removed by annealing at 220°C for 5 min. The Mar165 CCD detector had a pixel size of 79 µm. The size of the X-ray beam was 0.35 × 0.41 mm, as determined from the beam profile. The wavelength of the X-ray beam was fixed at 0.124 nm and the sample-to-detector distance was set to 2500 mm. The exposure time was 30 s. To translate 2D-SAXS patterns to 1D scattering profiles, the *Fit2D* software (Hammersley, 2016 ▸) from the European Synchrotron Radiation Facility was employed.

## 3. Scattering in a lamellar system   

### 3.1. Scattering from a (00*l*) crystal plane   

**Figure 2:**
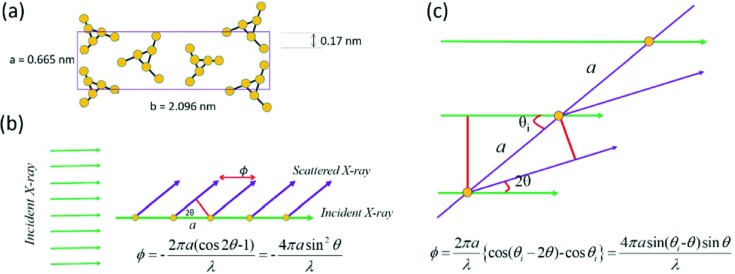
(*a*) The unit cell of α-iPP. (*b*) The phase difference between adjacent electrons on a (00*l*) crystalline plane parallel to the incident X-rays or (*c*) having an intersection angle θ_i_ with the incident X-rays.

**Figure 3:**
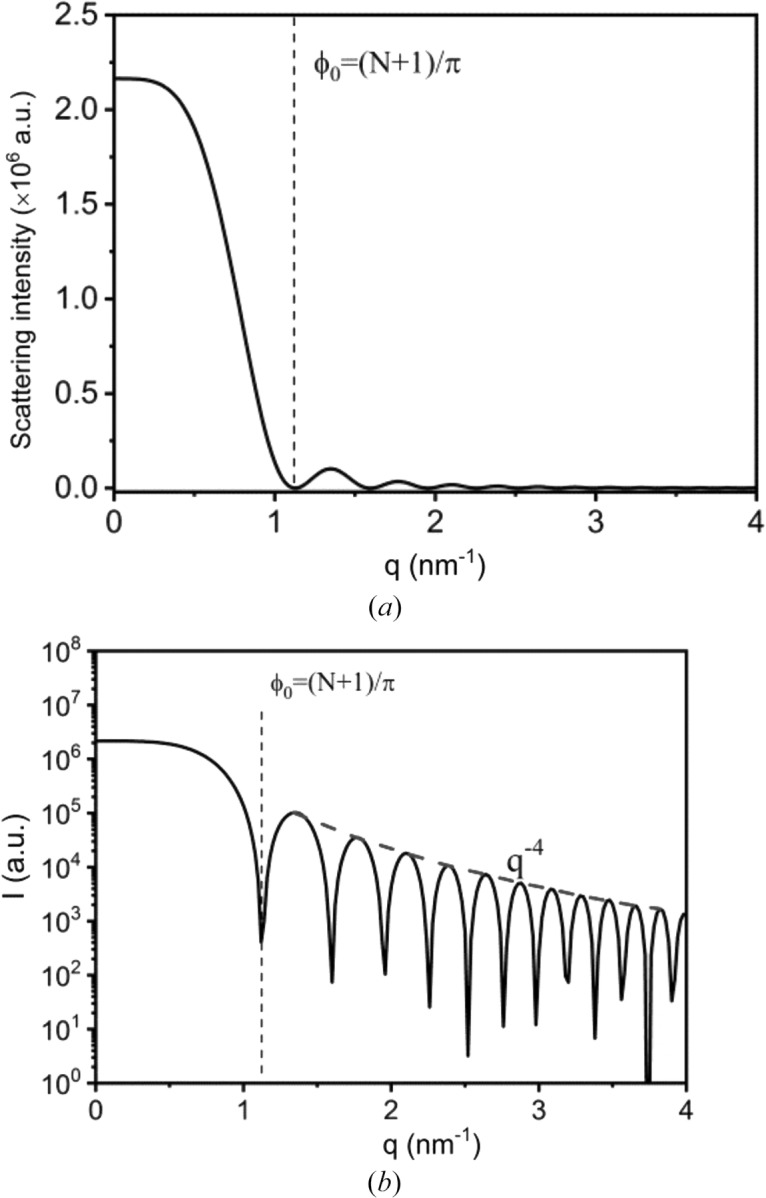
The scattering from the crystalline plane parallel to the incident X-rays in (*a*) linear and (*b*) logarithmic plots.

Before describing the scattering from a polymer spherulite, let us first determine the scattering from a (00*l*) crystal plane. To determine the scattering from a crystal plane, it is necessary to know the number of electrons on the plane (*N*) and the phase difference between adjacent electrons (ϕ): 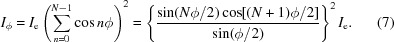
Here *I*_e_ is the scattering from a single electron. The phase difference between adjacent electrons can be determined with the following equation [see Fig. 2 ▸(*c*)]: 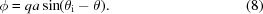
Here *a* is the average distance between adjacent electrons, and *q* = (4π sinθ)/λ. When the (00*l*) crystal plane is parallel to the incident X-rays, the phase difference is [see Fig. 2 ▸(*b*)] 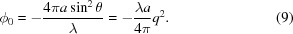
Using equations (7) and (9), the scattering from a crystal plane parallel to the incident X-rays can be determined. Given these two equations, the determination of the scattering intensity requires three values, *a*, *N* and λ. Estimating from the crystal cell of α-iPP, *a* is around 0.17 nm [see Fig. 2 ▸(*a*)]. The number of electrons is assumed to be 1471, since its corresponding size is 250 nm when *a* = 0.17 nm, which is the typical size of a lamella. The wavelength of the X-rays is assumed to be 0.124 nm. Fig. 3 ▸ shows the scattering profile from the (00*l*) crystal plane parallel to the incident X-rays. The profile can be divided into two regions. In Region I, the scattering is strong and decreases monotonically with *q*, while in Region II, the scattering is low and oscillates with *q*. The boundary between these two regions *q*_0_ corresponds to the phase difference 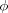 = π/(*N* + 1), since when this equality is met the scattering intensity becomes zero for the first time.

Such results can be understood straightforwardly. When 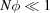, 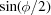, 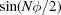 and 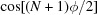 can be estimated using the following equations: 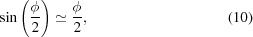


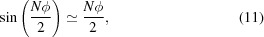


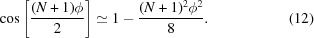
Therefore, equation (7) becomes
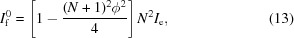
where 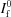 is the scattering at θ_i_ = 0. Combining with equation (9), equation (13) can be written further as 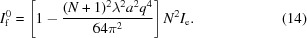
Given the above equation, when *q* = 0 nm^−1^, the scattering intensity is *N* ^2^ times *I*_e_, indicating fully constructive interference. Thus, strong scattering can be found. When *q* increases, the scattering intensity decreases rapidly with *q*.

When *N*ϕ is close to or greater than 1, equations (11) and (12) are no longer valid. Only equation (10) still holds. Thus, equation (9) becomes
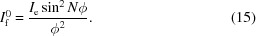
Here [(*N* + 1)ϕ]/2 is estimated to be *N*ϕ/2 in the derivation. Combining with equation (9), equation (15) can be written further as
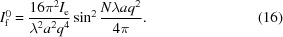
The scattering intensity has the same order of magnitude as *I*_e_, *i.e.* it is low. Meanwhile, due to the presence of the sine function, the scattering intensity oscillates with *q*. More importantly, the scattering intensity decreases with *q* ^−4^. This is the so-called Porod scattering.

Actually, equation (14) can be written in another form. (*N*+1)*a* is roughly equal to the lateral size of the crystal plane *l*_0_, therefore it can be written as
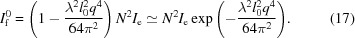
In the description of the scattering induced by the evanescent wave, equation (17) is employed to describe the scattering of the (00*l*) crystal plane parallel to the incident X-ray beam.

**Figure 4:**
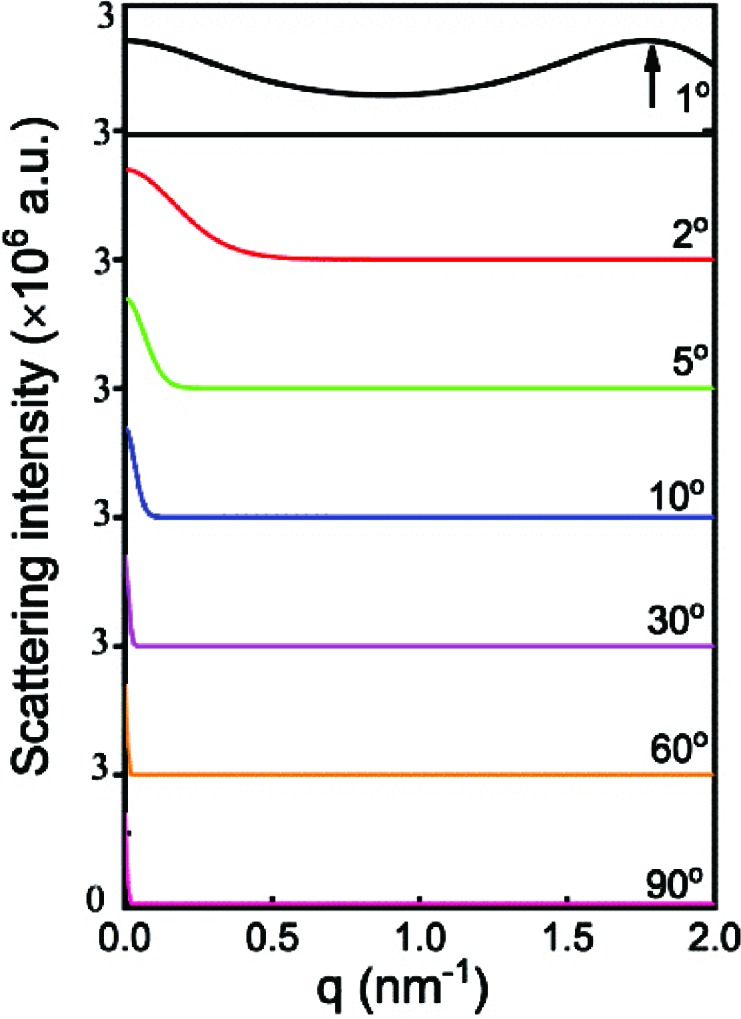
The scattering from the (00*l*) crystal planes at various incident angles. The numbers in the lower right corner of each curve represent the intersection angle of the crystal planes with the incident X-ray beam.

The scattering from an arbitrary crystal plane can be determined with equations (7) and (8). Fig. 4 ▸ shows the scattering at various incident angles. At θ_i_ = 1°, the scattering remains strong over the entire *q* range. A scattering peak can be seen at *q* = 1.78 nm^−1^. Its corresponding scattering angle is 1°, which is exactly equal to the incident angle. At higher incident angles, the scattering intensity decreases rapidly to a plateau, and the higher the incident angle, the earlier the scattering reaches the plateau.

This can be understood straightforwardly. As mentioned above, the boundary *q*_0_ corresponds to the phase difference 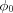 = π/(*N* + 1). Combining with equation (8), it can be found that 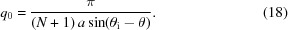
When θ_i_ > θ, the boundary *q*_0_ decreases with increasing incident angle.

It must be noted especially that not all crystal planes follow the Porod law in the high-*q* range. When θ_i_ ≫ θ, equation (8) can be estimated as
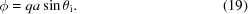
Combining with equation (15), the scattering in the higher *q* range can be written as
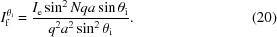
This scattering decays with *q* ^−2^ so it does not follow Porod’s Law. For a long time, Porod’s law has been applied to all planes (Albrecht & Strobl, 1996 ▸; Orench *et al.*, 2009 ▸; Verma *et al.*, 1996 ▸) but this is now shown to be a mistake. In fact, only the scattering from the crystal plane parallel to the incident X-ray beam satisfies Porod’s law.

A simple expression can also be obtained for the scattering from an arbitrary (00*l*) crystal plane. As mentioned above, when 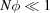, equation (7) can be simplified to equation (13). Combining with equation (8), it can be written as
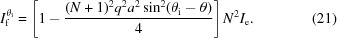
Furthermore, since (*N* + 1)*a* is roughly equal to *l*_0_, it can be written as
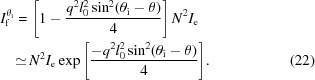
In the description of the scattering induced directly by the incident X-rays, equation (22) was employed to describe the scattering from the (00*l*) crystal plane.

### 3.2. Scattering of interfacial electrons induced by the evanescent wave   

**Figure 5:**
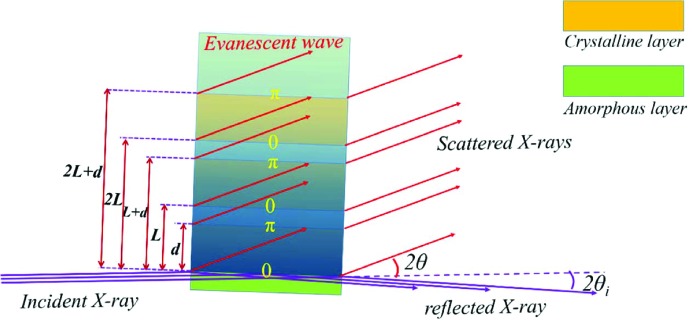
A schematic drawing of the scattering by interfacial electrons induced by the evanescent waves.

Let us look first at the scattering of the interfacial electrons involved in the evanescent wave. Fig. 5 ▸ shows a schematic drawing of the scattering, with a lamellar stack consisting of three lamellae as a model. When the incident angle θ_i_ is smaller than the critical total reflection angle for an amorphous/crystalline interface 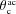, the incident X-ray beam is reflected totally at the first interface. Accompanied by total reflection, an evanescent wave forms at the interface, propagating downward and inducing electron scattering at other interfaces. Its intensity decays with the decay of the evanescent wave. Half-wave loss occurs at the second, fourth and sixth interfaces, since the X-ray beam enters the optically dense medium in normal incidence (Born & Wolf, 1999 ▸). The scattered X-rays interfere constructively, forming the scattering of the interfacial electrons involved in the evanescent wave.

To determine the intensity of the interference, we need the phases and amplitudes of the scattering at every interface. Since the evanescent wave decays exponentially with penetration depth, the scattering amplitude also decreases exponentially (Nishino *et al.*, 2000 ▸): 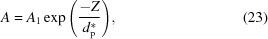
where *Z* is the distance of the interface from the first interface, 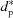 is the characteristic penetration depth and *A*_1_ is the scattering amplitude of the first interface. Normally, 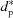 is around a few nanometres (Sakai *et al.*, 2005 ▸; Nishino *et al.*, 2000 ▸). Specifically, scattering amplitudes on different interfaces can be written as 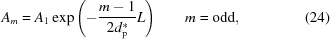


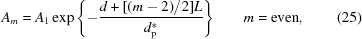
where *L* and *d* are the long period and the lamellar thickness, respectively. Here *m* is assumed to be 1 for the first interface. *A*_1_ can be estimated using equation (17),
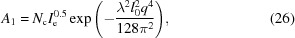
where *N*_c_ is the electron number of the first interface, or more accurately, the electron number of the first (00*l*) crystal plane.

Not considering the half-wave loss, the phase difference between two interfaces parallel to the incident X-ray beam can be determined by the following equation: 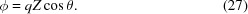
Nevertheless, since cosθ is close to 1 in the small-angle range, the above equation can be estimated as
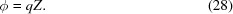
After considering the half-wave loss, the phases of scattered X-rays will be
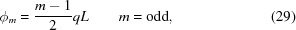


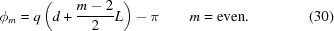
Using these phases and amplitudes, the overall scattering amplitude can be obtained by the following equation: 
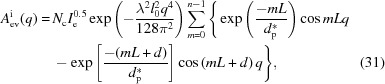
where *n* is the number of lamellae in the lamellar stack. It can be divided into two parts, the form factor 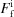 and the structure factor 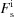, which are defined as
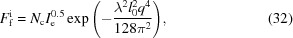


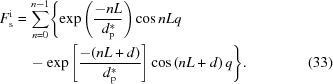
The form factor is the scattering amplitude from the first (00*l*) crystal plane and it includes information on the lateral size of the lamellar stack. The structure factor is the interference amplitude from parallel interfaces and it includes information on the lamellar thickness and the long period. As mentioned in the previous section, a crystalline plane parallel to the incident X-ray beam follows the Porod law [see equation (16)] in the high-*q* range. Therefore, the scattering of the interfacial electrons involved in the evanescent wave also follows the Porod law in the high-*q* range.

**Figure 6:**
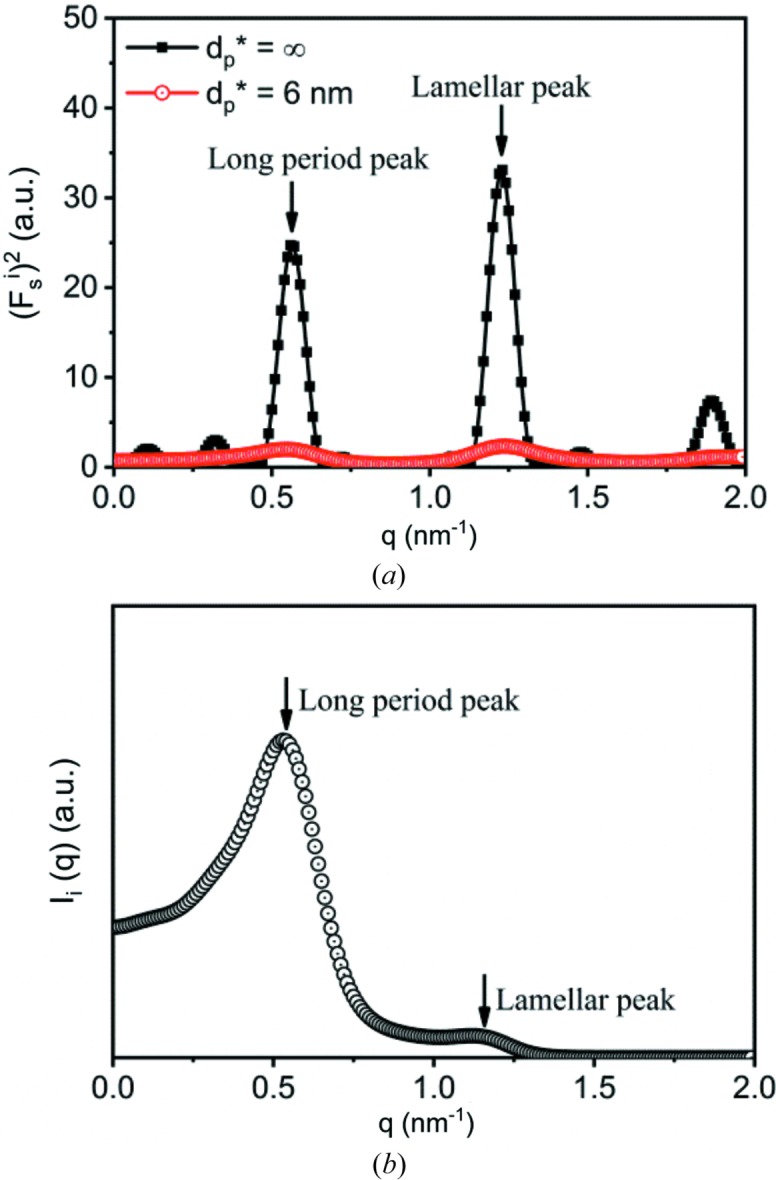
(*a*) Plots of 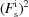 versus *q* with 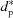 being infinite and 6 nm, respectively. (*b*) The scattering from the interfacial electrons involved in the evanescent wave. The long period, the lamellar thickness, the lateral size and the wavelength of X-rays were assumed to be 10.4 nm, 7.3 nm, 250 nm and 0.124 nm, respectively.

To see the scattering more clearly, the square of the form factor 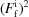 and the square of the structure factor 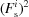 were determined with a lamellar stack consisting of three lamellae as a model. The lateral size, the characteristic penetration depth 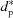, the long period and the lamellar thickness were assumed to be 250 nm, 6.0 nm, 10.4 nm and 7.3 nm, respectively. The scattering intensity is the product of 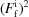 and 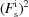. Fig. 6 ▸(*a*) shows 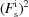 assuming 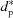 values of 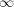 and 6 nm. Assuming 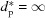 means no decay in intensity while the evanescent wave penetrates the lamellar stack. Many peaks can be seen in the 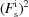 curve with 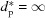. The highest peak should be from two interfaces a distance *d* apart, since estimating their phase difference using equation (30) gives a value roughly equal to 2π at *q* = 1.23 nm^−1^. The second-highest peak should be from two interfaces a distance *L* apart, since their phase difference is roughly equal to 2π at *q* = 0.57 nm^−1^ according to equation (29). Hereinafter, we call the highest and second-highest peaks the lamellar peak and the long-period peak, respectively. Other small scattering peaks can also be seen. Nevertheless, after considering the decay of the evanescent wave, these small peaks disappear, while only the long-period and lamellar peaks are left [see Fig. 6 ▸(*a*)]. Fig. 6 ▸(*b*) shows the scattering intensity. The long-period peak remains strong while the lamellar peak becomes negligible. This can be attributed to 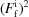. As seen in Fig. 3 ▸(*a*), only for small *q* does 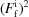 remain strong. With increasing *q*, 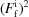 decreases rapidly. A smaller 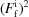 leads to a weaker lamellar peak.

### 3.3. Scattering of bulk electrons induced by the evanescent wave   

A similar method can be employed to determine the scattering of the bulk electrons involved in the evanescent wave. Nevertheless, two differences need to be noted. First, no half-wave loss exists in the scattering of the bulk electrons. As is known, the half-wave loss only occurs at the interface (Born & Wolf, 1999 ▸). Therefore, the phase of the scattering of the bulk electrons at the first plane can be determined directly from equation (28). Second, the scattering of the bulk electrons changes not only with the distance *Z*, but also with the electron densities. The electron densities in the amorphous layer and the crystalline layer are different, which will also affect the scattering intensity. The electron density at the interface remains unchanged. Thus, it is necessary to pay special attention to the location of the plane in determining the scattering amplitude, whether it is in the crystalline layer or the amorphous layer. Using equation (26), it is easily seen that, for a crystalline layer, its scattering amplitude is
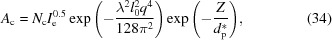
while for an amorphous layer its scattering amplitude will be
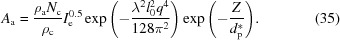
This is because in the amorphous layer, the number of electrons becomes 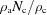.

With these phases and amplitudes, the interference amplitude of the bulk electrons in amorphous (*A*_ba_) and crystalline (*A*_bc_) layers can be determined separately: 
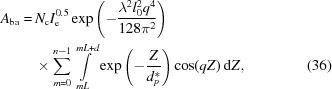


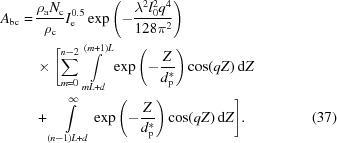
Here it is assumed that the last amorphous layer has an infinite thickness (see Fig. 5 ▸). The overall scattering amplitude is their sum: 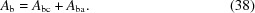
For convenience, we assume that the respective structure factors of the bulk electrons in the amorphous (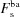) and crystalline layers (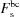) are
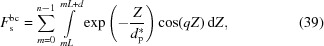


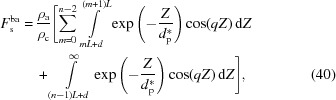
and they have the same form factor,
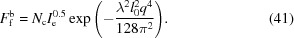
Thus, equation (38) can be written as
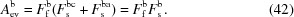
The overall structure factor of the bulk electrons 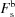 is the sum of 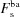 and 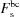.

To obtain the analytical solution of the structure factor, let us determine the integration in equation (39) first. To determine it conveniently, an imaginary part is added to the right-hand side: 
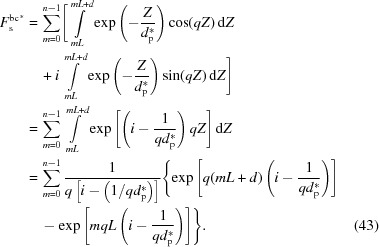
The real part is the structure factor of the crystalline layers, which is
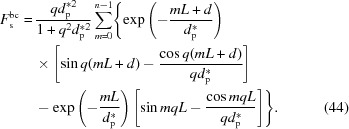
The integration in equation (40) can be also determined by the above method. The obtained structure factor of the amorphous layers is
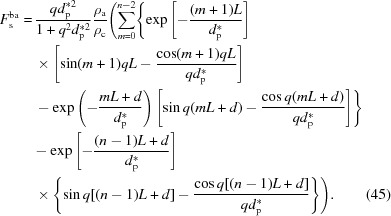
Thus, the overall structure factor of the bulk electrons 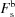 is
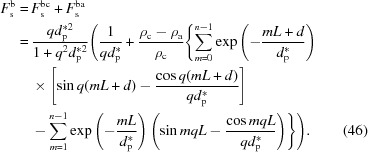
This can be divided into two parts: the first part is from the decay of the evanescent wave, and the second part is from the electron-density difference between the amorphous and crystalline layers.

**Figure 7:**
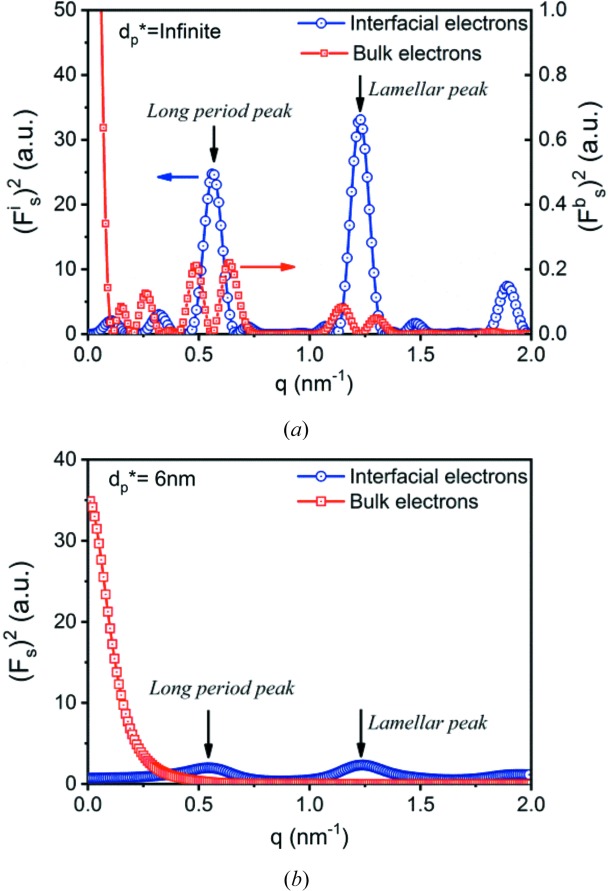
Plots of the square of the structure factors of the interfacial and bulk electrons assuming 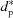 is (*a*) infinity and (*b*) 6 nm.

To see the structure factors more clearly, they were also determined with the same lamellar stack (Fig. 7 ▸). The long period, the lamellar thickness, the relative density difference between crystalline and amorphous iPP, and the wavelength of the X-rays were assumed to be 10.4 nm, 7.3 nm, 0.08 (Piccarolo *et al.*, 1992 ▸) and 0.124 nm, respectively. Let us look at 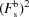 with 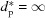 first. Assuming 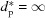 means that the intensity of the evanescent wave remains unchanged during its passage through the lamellar stack. For comparison, the square of the structure factor of the interfacial electrons 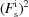 is also plotted in Fig. 7 ▸(*a*). An interesting finding is that double peaks are observed, rather than the single peaks that the bulk electrons form, and these are located around the long period and lamellar peaks from interfacial electrons. This is an important difference between the scattering from the bulk electrons and the interfacial electrons. Due to the smaller density difference, the double peaks have only 1% of the intensity of the long-period peak and the lamellar peak.

After considering the decay of the evanescent wave, all double peaks disappear [see Fig. 7 ▸(*b*)]. Only the first part of the structure factor can be seen in the plot. This is different from the structure factor of the interfacial electrons involved in the evanescent wave, where though the small peaks disappear, the two biggest peaks (the long-period peak and the lamellar peak) are still present.

**Figure 8:**
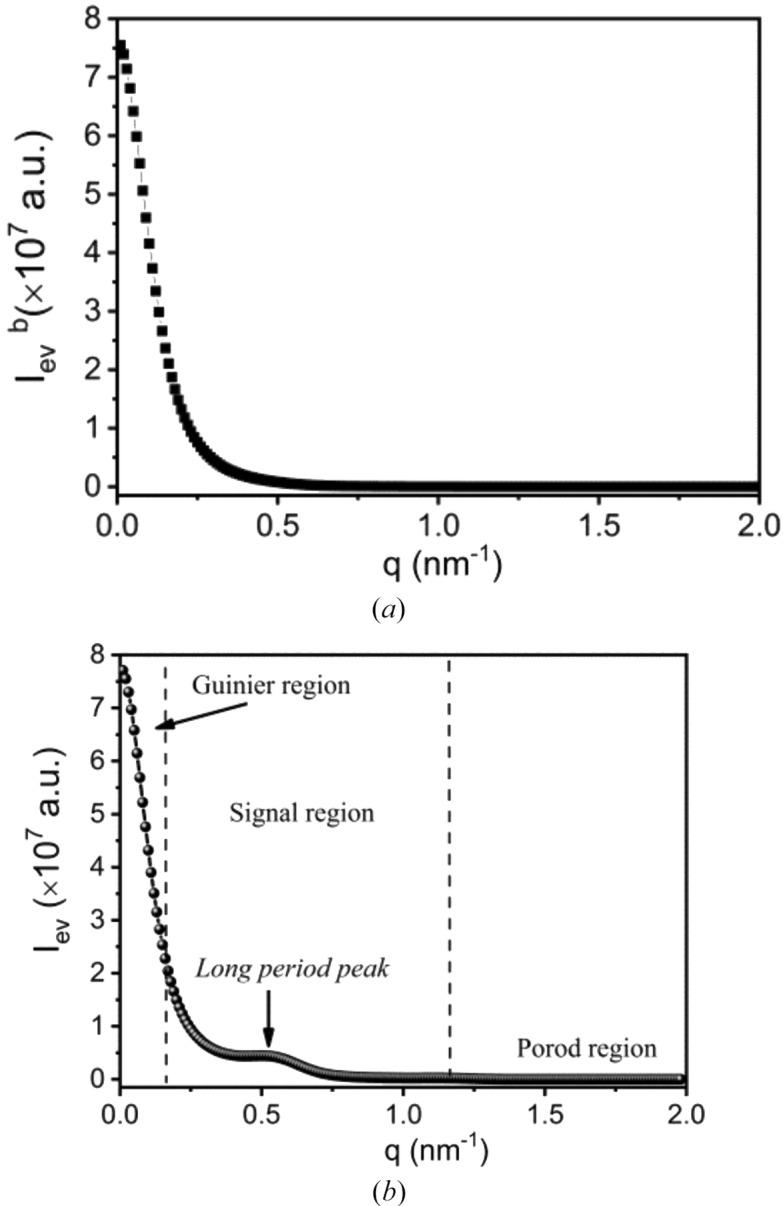
(*a*) The scattering of the bulk electrons involved in the evanescent wave (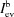) and (*b*) the overall scattering induced by the evanescent wave in a lamellar stack (*I*_ev_). The long period, the lamellar thickness, the relative density difference between crystalline and amorphous iPP, the lateral size, and the wavelength of the X-rays were assumed to be 10.4 nm, 7.3 nm, 0.08, 250 nm and 0.124 nm, respectively.

With the form factor and the structure factor, the scattering of the bulk electrons induced by the evanescent wave can be determined by equation (42). Fig. 8 ▸(*a*) shows the scattering of the bulk electrons involved in the evanescent wave. It differs from the scattering of the interfacial electrons in that no peak can be seen. The scattering decreases monotonically, similar to Guinier scattering (Guinier & Fournet, 1955 ▸). This can be explained straightforwardly. In the small-*q* range, the first term of the structure factor dominates the scattering [see equation (46)], and therefore the scattering intensity can be estimated as 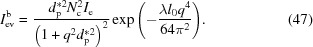
When 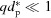, the two functions in equation (47) can be estimated as
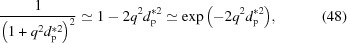


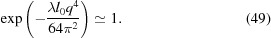
Here only the term of *q* ^2^ is retained. Therefore, equation (47) becomes
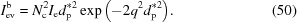
The intensity decays exponentially with *q* ^−2^ as in Guinier scattering. This indicates that the scattering of the bulk electrons involved in the evanescent wave is an origin of Guinier scattering.

Clearly, the scattering of the bulk electrons is always accompanied by that of the interfacial electrons. For a lamellar stack involved in the evanescent wave, its scattering includes the scattering from the bulk and interfacial electrons simultaneously. Fig. 8 ▸(*b*) shows the overall scattering induced by the evanescent wave from a lamellar stack (*I*_ev_). The scattering profile is similar to an observation in real SAXS measurements. We can divide it into three regions. In the small-*q* range, the scattering of the bulk electrons dominates the scattering, which follows the Guinier law [see equation (50)]. In the intermediate-*q* range, the long-period peak can be observed. In the high-*q* range, the scattering of the interfacial electrons dominates the overall scattering, which follows the Porod law (see Section 3.2). Therefore, these three regions can be called the Guinier region, the signal region and the Porod region, respectively.

### 3.4. Scattering induced directly by incident X-rays   

**Figure 9:**
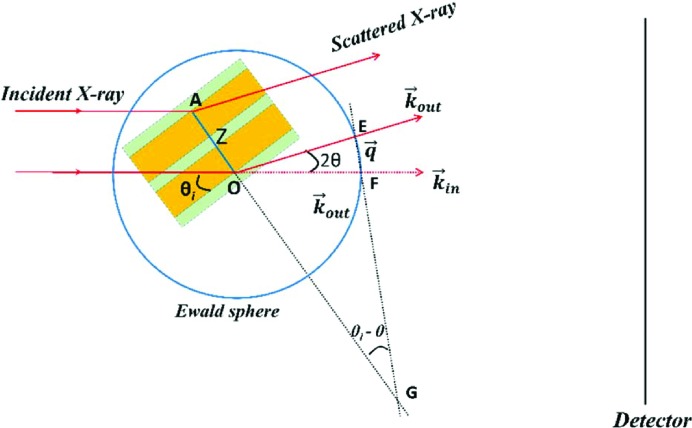
A schematic drawing of the scattering induced directly by the incident X-rays.

Lastly, let us look at the scattering induced directly by the incident X-rays. A schematic diagram for the scattering is shown in Fig. 9 ▸. Compared with the above two sources of scattering, the X-ray amplitude does not decay so fast. The characteristic penetration depth (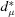) can be determined with the following equation (Sakai *et al.*, 2005 ▸): 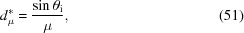
where μ is the linear absorption coefficient. For iPP, it has a value of 0.36 mm^−1^. According to this equation, the characteristic penetration depths at θ_i_ = 1° and 90° are 48 µm and 2.8 mm, respectively, much larger than that for total reflection. Scattering from the first interface can be described by equation (22). Therefore, the scattering amplitudes from the (00*l*) planes in crystalline (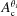) and amorphous (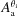) layers can be described separately by the following two equations: 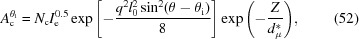


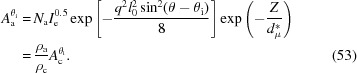
Also, no half-wave loss occurs in the scattering induced directly by the incident X-rays. Therefore, to determine the phase of an arbitrary (00*l*) plane, it is only necessary to know the phase difference from the first amorphous/crystalline interface. Since the scattering angle θ is not always equal to the incident angle θ_i_, we should employ equation (1) to determine the phase difference. From Fig. 9 ▸, it can be seen that the intersection angle between the wavevector and the distance is θ_i_ − θ. Therefore, the phase difference is
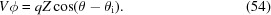
With these phases and amplitudes, the scattering of a lamellar stack induced directly by the incident X-ray beam can be determined as follows: 
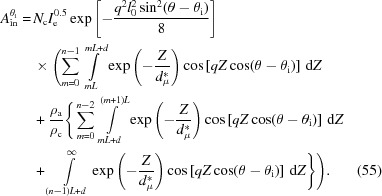
Note that here it is also assumed that the last amorphous layer has an infinite thickness as in the previous section. Thus we can similarly divide it into two parts. The first part can be defined as the form factor, denoted 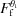, 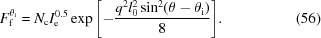
This is actually the scattering amplitude of the first interface. The second part can be defined as the structure factor, denoted 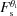,
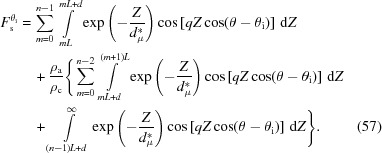
The scattering amplitude 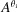 is the product of the form factor 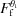 and the structure factor 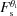. The integration in the structure factor can be also determined with the method in Section 3.3, which gives the result 
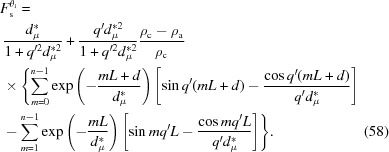
Here *q*′ = *q*cos(θ − θ_i_). As for the scattering of the bulk electrons involved in the evanescent wave, it can be also divided into two items. The first item is from X-ray absorption, and this is concentrated in the small-*q* range. The second item is proportional to the relative electron-density difference between the amorphous and crystalline layers, which is exactly one of the findings of Strobl & Schneider (1980 ▸). It is concentrated in the high-*q* range.

Compared with the lamellar stacks involved in total reflection, lamellar stacks which can be passed through directly by the incident X-rays are numerous. Assuming only one lamellar stack satisfying the total reflection condition in a spherulite, the number density of lamellar stacks (ρ_*n*_) will be
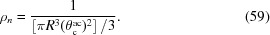
Here *R* is the radius of the spherulite. This means that around 1.1 × 10^7^ lamellar stacks exist in an iPP spherulite, for which the intersection angles with the incident X-ray beam are greater than the critical total reflection angle. For many such lamellar stacks, the interference between them must be considered.

**Figure 10:**
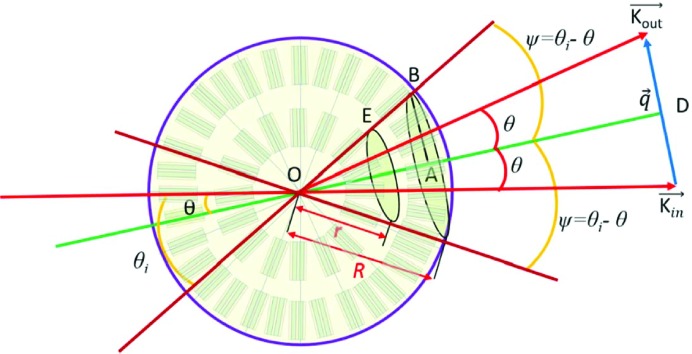
The scattering from lamellar stacks in a spherulite.

To determine this interference, it is necessary to know the phases and amplitudes of all lamellar stacks: 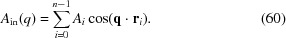
Here *A_i_* and **r**_*i*_ are the scattering amplitude and position vector, respectively, of a lamellar stack *i*. Assuming all lamellae in the spherulite grow along the radial direction, the phase can be determined as follows: 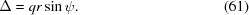
Here *r* is the distance between the lamellar stack at point *E* and the spherulitic centre *O*, and ψ is the intersection angle between **q** and **r**, which is equal to θ_i_ − θ (see Fig. 10 ▸). It is easy to see that if point *E* rotates along the radius *OA*, all lamellar stacks on the circle have the same phase.

From equations (56) and (58) it can be found that all lamellar stacks on the circle have the same form factor and almost the same structure factor. The small difference in the structure factor is from the difference in 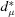. Nevertheless, because of the higher 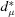, the structure factors have similar values, roughly equal to
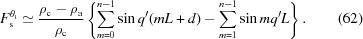
Having the same form factor and similar structure factors leads to almost the same scattering amplitude. We can thus simplify and assume that they have same scattering amplitude 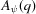. If we integrate first along the radius *OB* and then along ψ, the overall scattering induced directly by the incident X-rays in the spherulite can be determined,
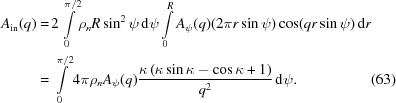
Here κ = *qR*sinψ.

At a small lateral size, a single-crystal plane can form strong scattering over a broad θ_i_ range [see Fig. 1 ▸(*b*)]. This means that lamellar stacks could also form strong scattering over a broad θ_i_ range. It can be assumed that lamellar stacks in the range of 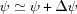 have the same scattering amplitude, such that the above integration can be estimated as
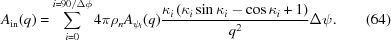
Here κ_*i*_ = *qR*sin(*i*Δψ).

At an infinite lateral size, the crystal plane only has strong scattering near θ = θ_i_ [see Fig. 1 ▸(*b*)]. Equation (56) can be rewritten as
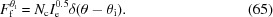
Therefore, the integration in equation (63) can be reduced to
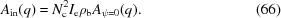
Here ρ_b_ is the number of lamellar stacks satisfying the Bragg condition in a spherulite at a given wavevector. This means that only the lamellar stacks satisfying the Bragg condition make a contribution to the scattering. For 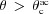, the above equation can be written as 
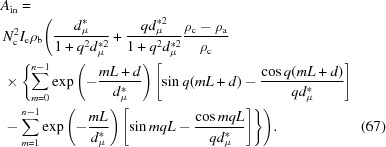
Note that *q*′ has changed to *q* because θ = θ_i_. This gives most of the scattering in the small-angle range. For example, for iPP, it can give the scattering at *q* > 0.06 nm^−1^ when λ = 0.124 nm. This is because the wavevector at 0.06 nm^−1^ corresponds to θ at 0.034°, which is exactly equal to the critical total reflection angle at the amorphous/crystalline interface of iPP. The scattering induced by the evanescent wave does not need to be considered, because only when 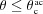 is the scattering that induced by the evanescent wave. In common SAXS measurements, the scattering below *q* = 0.1 nm^−1^ is normally obstructed by the beamstop.

To see the scattering induced by incident X-rays clearly, scattering profiles in a lamellar system with finite and infinite lamellar lateral sizes were determined, assuming a long period, lamellar thickness, linear absorption coefficient and relative electron-density difference of 10.4 nm, 7.3 nm, 0.36 mm^−1^ and 0.08, respectively. For the lamellar system with limited lateral size, the lamellar lateral size was assumed to be 250 nm.

**Figure 11:**
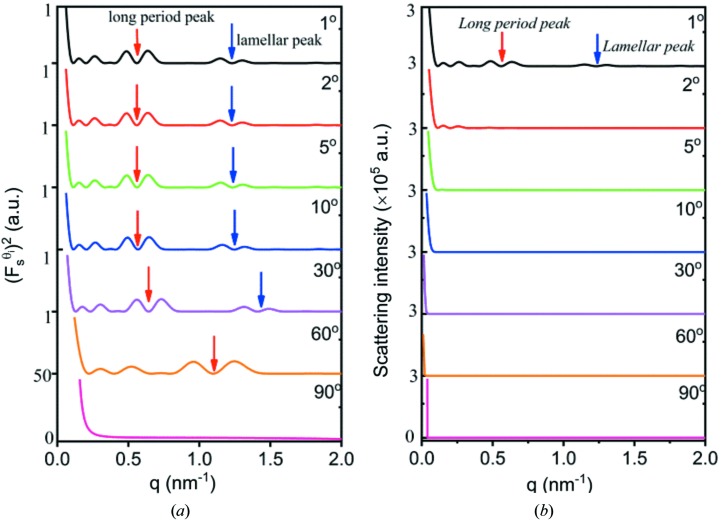
(*a*) Plots of 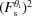 and (*b*) plots of 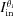 at various incident angles (1, 2, 5, 10, 30, 60 and 90°). The long period, the lamellar thickness, the linear absorption coefficient, the lateral size and the relative electron difference were assumed to be 10.4 nm, 7.3 nm, 0.36 mm^−1^, 250 nm and 0.08, respectively.

Let us first look at the scattering from a lamellar stack with finite lateral size. The form factor and structure factor can be obtained by equations (56) and (58), respectively, and the scattering amplitude is their product. Fig. 11 ▸(*a*) shows 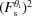 at various incident angles. The form factor is similar to that in Fig. 4 ▸, so it is not shown here. A strong structure factor can be found around *q* = 0, which should be from X-ray absorption. In the high-*q* range, double peaks can be seen, which should be from the electron-density difference between the amorphous and crystalline layers. With increasing incident angle, these peaks shift constantly to a higher *q* range. This can be understood easily from equation (58). For a fixed *q*′, *q* increases with increasing θ_i_ when θ_i_ > θ. Fig. 10 ▸(*b*) shows 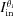 at various incident angles. The scattering intensity is concentrated in the small-*q* range. Outside the small-*q* range, only at θ_i_ = 1° do double peaks still exist [see Fig. 11 ▸(*b*)]. Obviously, the absence of the double peaks at other incident angles is due to weak form factors (see Fig. 4 ▸).

**Figure 12:**
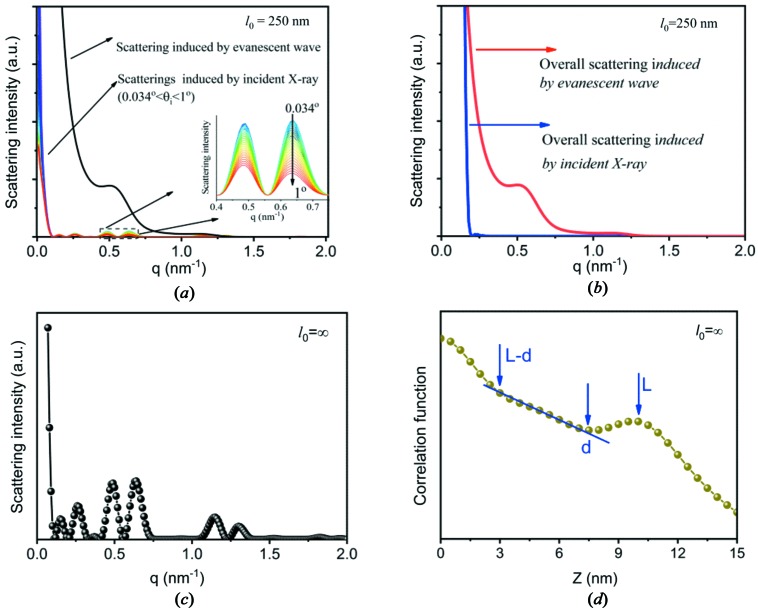
(*a*) The scattering from lamellar stacks with θ_i_ = 0–1°. The scattering of the lamellar stack with θ_i_ = 0 is induced by the evanescent waves, while the scattering from the lamellar stacks with θ_i_ = 0.034–1° is induced directly by the incident X-rays. (*b*) The overall scattering induced by the incident X-ray beam and the evanescent waves, respectively, in a spherulite with a lamellar lateral size of 250 nm. (*c*) The overall scattering from a lamellar system with infinite lateral size. (*d*) The Fourier transform result for the scattering in panel (*c*).

At a smaller lateral size, lamellar stacks with similar incident angles indeed have similar intensities. To show this point, we determined the scattering profiles of lamellar stacks in the range of θ_i_ = 0–1°. Assuming θ_i_ for the first lamellar stack is 0° and θ_i_ increases by 0.034° every time, there are therefore 29 lamellar stacks. From the second lamellar stack on, the scattering will be induced directly by the incident X-rays. Fig. 12 ▸(*a*) shows scattering profiles induced by incident X-rays from individual lamellar stacks. The profiles have similar intensities and peak positions, as shown in the inset. The scattering from the first lamellar stack is also plotted in Fig. 12 ▸(*a*). For first lamellar stack, θ_i_ = 0, and therefore its scattering is induced by the evanescent wave. The scattering is much stronger than that induced by the incident X-ray beam. The strong scattering is due to two factors, fast decay of the evanescent wave and half-wave loss (see Sections 3.2 and 3.3), while the low intensity in the scattering induced by the incident X-rays is due to the small electron-density difference.

For a lamellar system with limited lamellar lateral size, the overall scattering intensity at a finite lateral size can be determined by equation (64), assuming a spherulite radius of 100 µm and Δψ = 1°. Compared with the scattering from individual lamellar stacks, the scattering in the small-*q* range is increased because of constructive interference, while the scattering in the high-*q* range is reduced because of destructive interference [see Fig. 12 ▸(*b*)]. As mentioned above, the scattering in the small-*q* range is from X-ray absorption, while the scattering in the high-*q* range is from the electron-density difference. This means that after interference, only the scattering from X-ray absorption remains. The scattering induced by the evanescent wave in the spherulite is also plotted in Fig. 12 ▸(*b*). Since only one lamellar stack was assumed to be involved in the evanescent wave in the spherulite, the scattering from this lamellar stack is the overall scattering induced by the evanescent wave. The scattering induced by the evanescent wave in the spherulite is much stronger than that induced by incident X-rays in the *q* range from 0.2 to 1.3 nm^−1^. This indicates that the scattering induced by the evanescent wave is the main origin of SAXS in a lamellar system with limited lateral size.

At an infinite lateral size, the overall scattering can be obtained by equation (67). Fig. 12 ▸(*c*) shows the scattering at 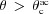, which is induced directly by incident X-rays. Double peaks can be seen, which are due to the electron-density difference between the crystalline and amorphous layers. The scattering induced by the evanescent wave is not shown here since it only appears below *q* = 0.06 nm^−1^, as mentioned above. Not considering X-ray absorption, equation (67) is the solution of the integration in equation (4). Carrying out the Fourier transform for the scattering we obtain the correlation function 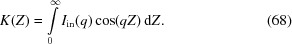
Fig. 12 ▸(*d*) shows the Fourier transform result. A linear region can be seen before the long-period peak. The ends correspond to the amorphous and lamellar thicknesses, respectively. This is exactly the main conclusion drawn by Strobl & Schneider (1980 ▸) and shows that our determination of the scattering in a lamellar system with infinite lamellar lateral size is correct.

From the above results, it can be found that there are two totally different scattering models. At an infinite lamellar lateral size, the scattering is mainly that induced directly by the incident X-rays. The scattering is mainly from the electron-density difference between the amorphous and crystalline layers. At a limited lateral size, the scattering is largely that induced by the evanescent wave. The scattering is mainly from the ordered arrangement of (00*l*) crystal planes in a lamellar stack. This point is similar to the viewpoint proposed recently by Konishi *et al.* (2018 ▸).

Here we would like to discuss briefly the condition that the scattering induced by incident X-rays dominates the scattering. As seen above, the factor leading to the negligibility of *I*_in_ is destructive interference. Clearly, if in Δψ there exists only one lamellar stack in a spherulite, there will be no destructive interference. This means the arc length corresponding to Δψ in the spherulite needs to be smaller than the thickness of lamellar stack *T*, 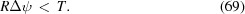
Δψ can be estimated from the FWHM of the scattering of the (00*l*) crystal plane. According to equation (56), Δψ is equal to 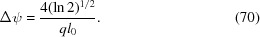
Therefore, the lateral size needs to satisfy the following equation: 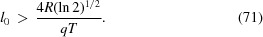
Assuming the thickness of the lamellar stack is 30 nm and the radius of the spherulite is 100 µm, the lateral size needs to be greater than 11 µm at *q* = 1 nm^−1^, and 0.4 µm at 2θ = 30°. This indicates that for a lamellar system of limited lamellar lateral size, destructive interference needs to be considered in SAXS but not in WAXD. The SAXS from a real polymer lamellar system arises mostly from the scattering induced by the evanescent wave.

This conclusion sounds like a fantastic tale at first. The SAXS signal arises only from a minority of the lamellar stacks involved in total reflection. However, the idea will not seem strange after comparing with WAXD. As is known, the Bragg condition is like a band-pass filter, in that only crystal planes satisfying the Bragg condition make a significant contribution to WAXD (Bragg, 1913 ▸). The bandwidth can be determined using equation (70). For a lamellar crystal with a lateral size of 0.4 µm, Δψ is equal to 0.031° at 2θ = 30°, which is even smaller than the critical total reflection angle of iPP at the amorphous/crystalline interface. This means that WAXD also has contributions from a minority of lamellar stacks at a given wavevector. At small *q*, the role of the Bragg condition as a band-pass filter is out of action. Instead, the total reflection plays the role of band-pass filter. It excludes most of the lamellar stacks, avoiding destructive interference.

## 4. Preliminary experimental evidence   

In the above section, a surprising conclusion was drawn. For a lamellar system with a finite lateral size (a few hundred nanometres or less), SAXS is mainly induced by the evanescent wave. The scattering induced directly by the incident X-rays is negligible because of destructive interference. It exists only in the small-*q* range, which is mainly from X-ray absorption. Intuitively, this still seems impossible. To check whether it is right or not, the best judgement undoubtedly is real scattering from a crystallized polymer. In this study, we made a preliminary check with iPP scattering profiles, which were obtained during isothermal crystallization at 130°C after removal of thermal history at 220°C. It was reported that at a lower temperature, iPP formed a lamellar two-phase structure (Zhu *et al.*, 2001 ▸).

**Figure 13:**
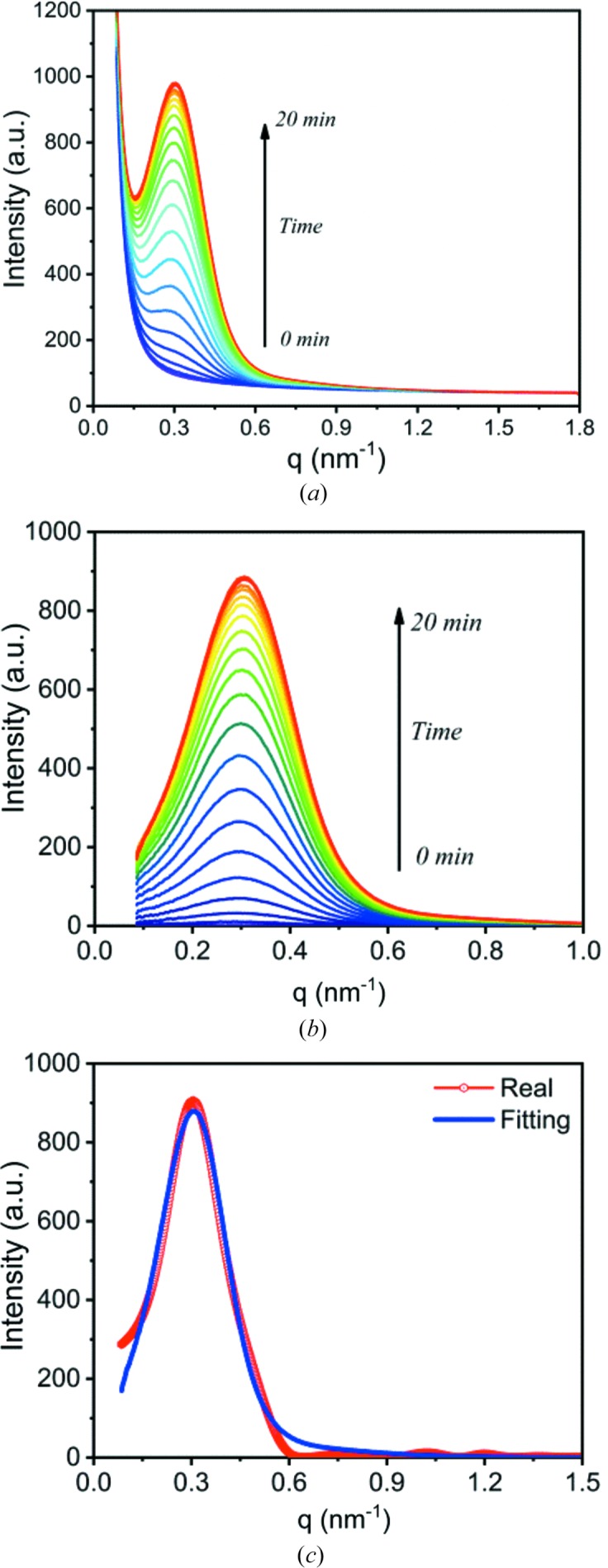
(*a*) The change in the scattering intensity during isothermal crystallization at 130°C after removal of thermal history at 220°C for 5 min. (*b*) Scattering profiles after subtracting the first scattering profile at *t* = 0 min. (*c*) The fit to the scattering profile after complete crystallization.

Fig. 13 ▸(*a*) shows the scattering profiles. Only a single peak can be seen, which implies that the scattering is probably from interfacial electrons. As seen in Sections 3.3 and 3.4, only interfacial electrons can form single peaks in the scattering profile; the bulk electrons in the alternately arranged amorphous and crystalline layers form double peaks in the scattering profile.

To check further, it is necessary to know more information, for example, the lateral size and the characteristic penetration depth. If the scattering were indeed induced mainly by the evanescent wave, the lateral size should be short and the characteristic penetration depth should be only a few nanometres. As discussed in Section 3.4, it is only at a limited lateral size that the scattering induced by the evanescent wave dominates the profile. A rapid decay in the X-ray intensity on the nanoscale is the signature of the evanescent wave.

Clearly, if we know the scattering of the interfacial electrons involved in the evanescent wave 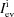, such information can be obtained by fitting equation (31). The question is how to separate 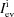 from the real scattering. As is known, there are other sources of scattering in polymer systems, for example the scattering induced by the incident X-rays *I*_in_ and the scattering of the bulk electrons involved in the evanescent wave 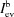. We know from Section 3.4 that *I*_in_ arises mainly from X-ray absorption at a finite lateral size. Therefore, *I*_in_ can be displaced roughly by the melt scattering before isothermal crystallization *I*_melt_. We also know from Section 3.3 that in the intermediate-*q* range, the scattering of interfacial electrons dominates the scattering induced by the evanescent wave. Therefore, 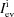 can be obtained roughly by the following equation: 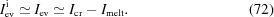
Here *I*_cr_ is the scattering from the crystallized sample. This has another benefit. In real scattering, there are other sources of scattering, for example, air scattering, liquid scattering (the scattering from the amorphous polymer between lamellar stacks) (Verma *et al.*, 1996 ▸) and the scattering from optical windows, which will not change during isothermal crystallization. Therefore, subtracting the melt scattering can clearly remove these scattering sources.

Fig. 13 ▸(*b*) shows scattering profiles after subtracting the first profile. The scattering intensity is concentrated in the intermediate-*q* range (0.1–0.6 nm^−1^). Over this small *q* range, it can be fitted with equation (31). Fig. 13 ▸(*c*) shows the fitting result for the scattering after complete crystallization. The fitted curve almost overlaps with the real scattering profile. The structural parameters obtained by the fit, *L*, *d*, *l*_0_ and 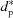, were 21.5 nm, 8.3 nm, 759.6 nm and 8.3 nm, respectively. These values are basically in accordance with reported values (Li *et al.*, 1999 ▸; Yamada *et al.*, 2003 ▸; Sakai *et al.*, 2005 ▸; Li *et al.*, 2006 ▸; Kailas *et al.*, 2007 ▸; Mani *et al.*, 2016 ▸; Lu *et al.*, 2017 ▸), indicating that the fit is reliable. Two important parameters are the lateral size and 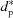. The 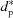 value of 8.3 nm indicates the existence of the evanescent wave, while the lateral size of 759.6 nm indicates the existence of destructive interference. From these two parameters, it can safely be concluded that the scattering in crystallized iPP is indeed induced mainly by the evanescent wave.

## 5. Conclusions   

To summarize, at a finite lamellar lateral size, the scattering induced by evanescent waves, especially the scattering from interfacial electrons, is most likely to be the main origin of SAXS in polymer lamellar systems. It can form a similar interference pattern to that observed in real scattering: a Guinier region in the small-*q* range, a signal region in the intermediate-*q* range and a Porod region in the high-*q* range. On the other hand, the scattering induced directly by the incident X-rays is negligible because of destructive interference between the lamellar stacks in a spherulite. It exists only in the small-*q* range, where it is mainly due to X-ray absorption. The measured scattering from iPP has demonstrated such a possibility.

Based on these findings, we would like to propose a new SAXS model for real polymer lamellar systems, where the lamellar crystals have dimensions of only a few hundred nanometres in lateral size. In polymer spherulites there are a great number of lamellar stacks. For most of these lamellar stacks, the incident X-rays will pass through them directly, without inducing strong scattering. Only for a minority of lamellar stacks can strong scattering be induced, when the amorphous/crystalline interfaces are almost parallel to the incident X-rays. When the X-ray beam reaches such lamellar stacks, it will be totally reflected at the first interface. Accompanied by total reflection, an evanescent wave forms at the first interface and propagates downward, inducing electron scattering. This scattering, especially that from interfacial electrons, interferes constructively, forming a strong SAXS signal. Finally, it needs to be noted that the model also assumes that the SAXS signal also arises from the electron-density difference, since if no density difference exists, reflection will not occur, let alone total reflection.

## Supplementary Material

Additional theoretical background. DOI: 10.1107/S2052252519011035/ed5020sup1.pdf
