# Supplementary material for: A new small-angle X-ray scattering model for polymer spherulites with a limited lateral size of the lamellar crystals
Source: IUCrJ. 2019 Aug 31;6(Pt 5):968–83. doi: 10.1107/S2052252519011035 (PMC6760438; doi:10.1107/S2052252519011035)
Supplement: Supplementary file 1 [file m-06-00968-sup1.pdf]

IUCrJ

Volume 6 (2019)

Supporting information for article:

New Small-Angle X-ray Scattering Model for Polymer Spherulite with Limited Lateral Size of Lamellar Crystals

Xiangyang Li, Jianjun Ding, Yanping Liu and Xingyou Tian

S1. Contributions of Crystal Planes of Different Orientations to Small-Angle X-Ray Scattering at *q* = 0.5 nm^{-1} and Wide-Angle X-Ray Diffraction at *2θ* = 30°

To determine a scattering from a crystal plane, it needs the number of electrons ***N*** and the phase difference between adjacent electrons ϕ:

$$I_\phi = I_e(\sum_{n=0}^{N-1}\cos n\phi)^2 = (\frac{\sin\frac{N\phi}{2}\cos\frac{(N+1)\phi}{2}}{\sin\frac{\phi}{2}})^2 I_e \qquad \text{(S1)}$$

Here I_e is the single-electron scattering intensity. The phase difference ϕ can be determined by the following equation (See Fig. S1b):

$$\phi = \frac{4\pi a \sin(\theta_i - \theta)\sin\theta}{\lambda} \qquad \text{(S2)}$$

Here θ_i is the intersection angle of the (*00l*) crystal plane with the incident X-ray, and *a* is the distance between adjacent electrons.

Estimating with the unit cell of α crystal of iPP, the average distance between adjacent electrons is 0.17 nm (see Fig. S1a). Therefore, for an iPP crystal plane parallel to incident X-ray ($\theta_i = 0$), phase difference of adjacent electrons is -0.024° for q = 0.5 nm^{-1}, and 66° for *2θ* = 30°. Phase differences at various incident angles were also determined at fixed *q* or *2θ*. The wave vector *q* in Fig. S2a is fixed to be 0.5 nm, while *2θ* is fixed to be 30° in Fig. S2b. When *q* is fixed to be 0.5 nm^{-1} (corresponding scattering angle θ = 0.28°), the phase difference increases from -0.024° to 0.4° when the incident angle increases from 0 to 5°. While *2θ* is fixed to be 30° (corresponding q = 26.2 nm^{-1}), the phase difference increases from -66.1° to 246.7° when the incident angle increases from 0 to 90°.

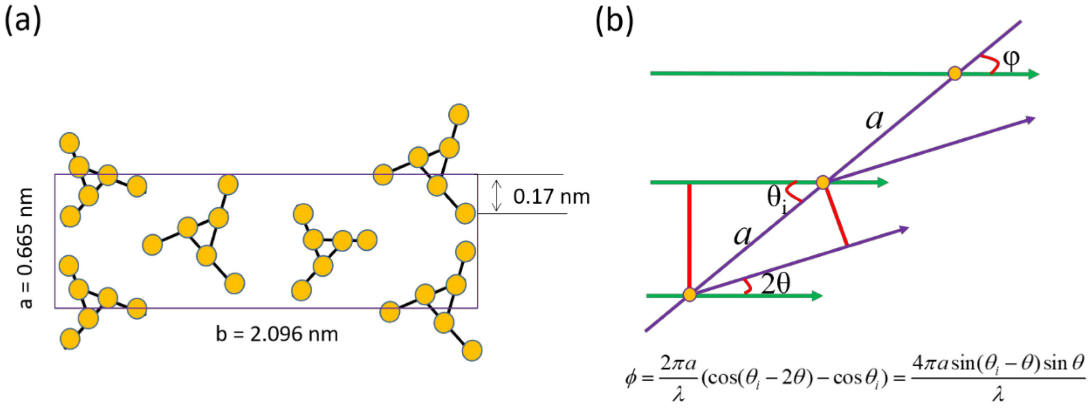

Figure S1 (a) A unit cell of α crystal of iPP. (b) Determination of the phase difference between adjacent electrons on a crystal plane having an intersection angle θ_i with the incident X-ray.

Scattering intensity from a crystal plane was also determined at fixed q or 2θ employing Eq. S1. In Fig. S2a and S2b, the electron number on the crystal plane was assumed to be 1471, since its corresponding lateral size is 250 nm, which is the typical size of a lamella. When q is fixed to be 0.5 nm^{-1} (corresponding scattering angle $\theta = 0.28^o$), it can remain strong scattering in a broad range of θ_i=0 - 1° because of small phase difference. While 2θ is fixed to be 30° (corresponding q = 26.2 nm^{-1}), it only can form strong scattering at $\theta_i = \theta = 15^o$. This is because large phase difference exists in adjacent electrons. Only at $\theta_i = \theta = 15^o$, the scatterings of the electrons have the same phase, forming fully constructively interference. This is so-called Bragg condition.

A scattering of a crystal plane with large lateral size was also determined. The lateral size was assumed to be 10 μm. Under larger lateral size, scattering intensity concentrates in narrow range similar to Bragg scattering (see Fig. S2c).

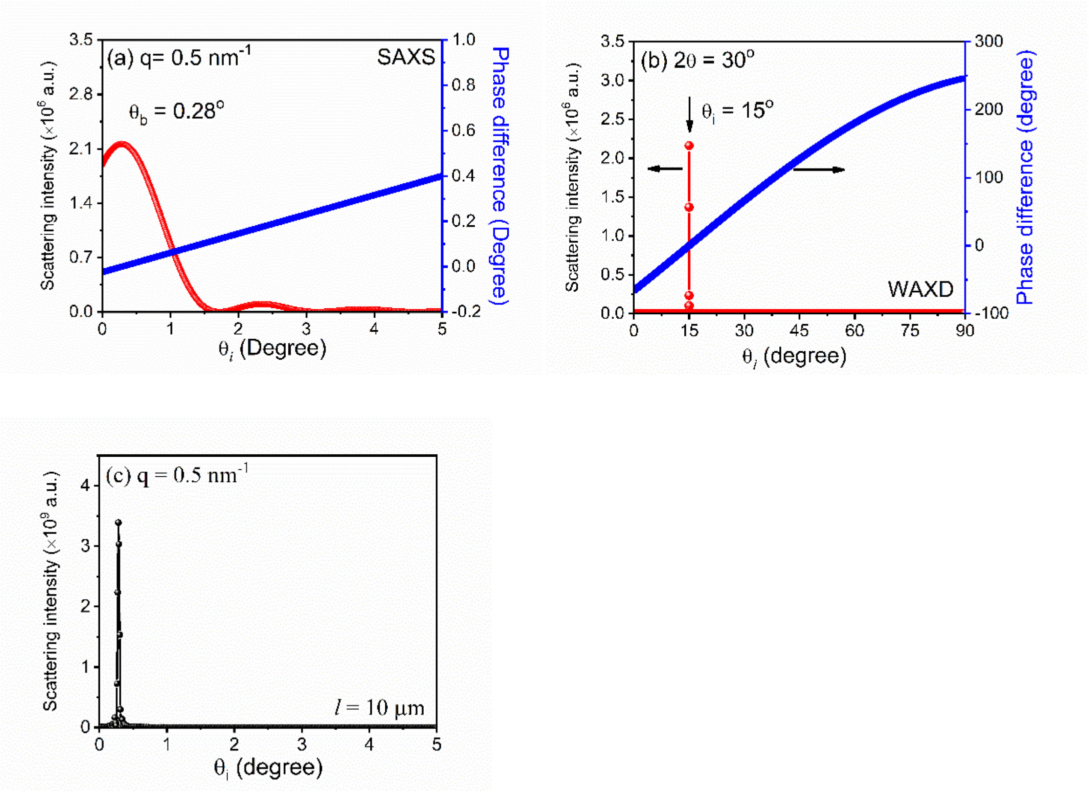

Figure S2. Scattering intensities and corresponding phase differences between adjacent electrons on a crystal plane at various incident angles under fixed q or 2θ. In (a) and (c), q is fixed to be 0.5 nm^{-1}, while in (b) 2θ is fixed to be 30°. The lateral size in (a) and (b) was assumed to be 250 nm, and the lateral size in (c) was assumed to be 10 μm. The average distance between adjacent electrons and the wavelength of X-ray in (a-c) were assumed to be 0.17 nm and 0.124 nm, respectively.

S2. Determination of Critical Total Reflection Angle on the Amorphous/Crystalline Interface of isotactic Polypropylene

The critical total reflection angle on the amorphous/crystalline interface can be determined with Fresnel Equation. As is known, for two mediums with refraction indices n_i, n_r, respectively, the incident angle (θ_i) and the refracted angle (θ_r) satisfy the following relationship:(Born & Wolf, 1999)

$$n_i \cos\theta_i = n_r \cos\theta_r \qquad \text{(S3)}$$

Let the amorphous layer be the incident medium, while the crystalline layer as the refractive medium, the refracted angle θ_r will be smaller than the incident angle θ_i. This is because amorphous polymer has a higher refraction index than crystalline polymer, which can be seen from the following equation:(Jeu, 2016)

$$n = 1 - \delta + i\beta = 1 - \frac{\lambda^2}{2\pi} r_e \rho + i\frac{\lambda}{4\pi}\mu \qquad \text{(S4)}$$

where r_e is Thompson scattering length of a single electron, λ is the wavelength of X-ray and μ is the linear absorption coefficient. From Eq. S4, it can be found that the higher the density, the lower the refraction index. Evidently, crystalline polymer (ρ_c) has a higher density than amorphous polymer (ρ_a), therefore, it has a lower refraction index.

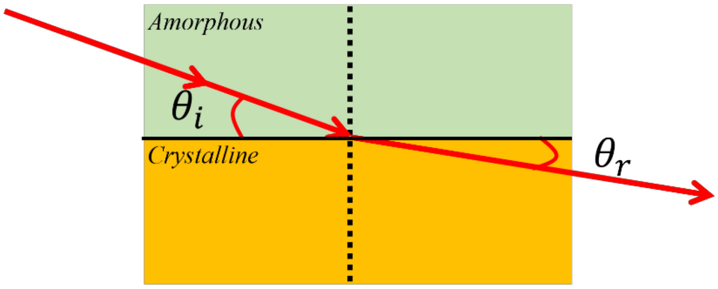

Figure S3. Refraction of X-ray on the amorphous/crystalline interface.

When $\theta_r = 0$, the incident angle is the critical angle for total reflection θ_c^{ac}. The Fresnel Equation becomes as:

$$\cos\theta_c^{ac} = \frac{n_r}{n_i} \qquad \text{(S5)}$$

Since $\theta_c^{ac} \ll 1$, $\cos\theta_c^{ac}$ can be estimated as $1 - \frac{(\theta_c^{ac})^2}{2}$. Meanwhile, estimating with Eq. S4, the refraction indices n_a, n_c are roughly equal to $1 - r_e\rho_a\lambda^2/2\pi$ and $1 - r_e\rho_c\lambda^2/2\pi$, respectively. Therefore, the critical total reflection angle can be estimated as:

$$\theta_c^{ac} = \sqrt{\frac{r_e\lambda^2}{\pi}(\rho_c - \rho_a)} \qquad \text{(S6)}$$

It was reported that the critical total reflection angle θ_{ap} on the vacuum/iPP interface was 0.12° for X-ray with λ = 0.124 nm, which was obtained with the following equation:(Sakai *et al.*, 2005)

$$\theta_c^{ap} = \sqrt{\frac{r_e \lambda^2}{\pi} \rho_c} \qquad \text{(S7)}$$

Combining Eq.S7, Eq. S5 can be written further as:

$$\theta_c^{ac} = \varphi_c^{ap} \sqrt{\frac{\rho_c - \rho_a}{\rho_c}} \qquad \text{(S8)}$$

As is known, the amorphous density of iPP is around 92% of crystalline density.(Piccarolo *et al.*, 1992) Thus, the critical total reflection angle on the amorphous/crystalline interface θ_c^{ac} is around 0.034^{o}.

References

Born, M. & Wolf, E. (1999). *Principle of Optics*. Cambridge: Cambridge University Press.

Jeu, W. H. d. (2016). *Basic X-ray scattering for soft matter*. Oxford: Oxford University Press.

Piccarolo, S., Saiu, M., Brucato, V. & Titomanlio, G. (1992). *Journal of Applied Polymer Science* **46**, 625-634.

Sakai, A., Tanaka, K., Fujii, Y., Nagamura, T. & Kajiyama, T. (2005). *Polymer* **46**, 1717-1717.
